# Socio-economic influences on anthropometric status in urban South African adolescents: sex differences in the Birth to Twenty Plus cohort

**DOI:** 10.1017/S1368980015000415

**Published:** 2015-03-11

**Authors:** Rebecca Pradeilles, Paula L Griffiths, Shane A Norris, Alison B Feeley, Emily K Rousham

**Affiliations:** 1 Centre for Global Health and Human Development, School of Sport, Exercise and Health Sciences, Loughborough University, Loughborough, Leicestershire, LE11 3TU, UK; 2 MRC/Wits Developmental Pathways for Health Research Unit, University of the Witwatersrand, Johannesburg, South Africa

**Keywords:** Socio-economic, Household, Neighbourhood, Anthropometric status, Adolescents, Urban, South Africa

## Abstract

**Objective:**

To investigate the associations of household and neighbourhood socio-economic position (SEP) with indicators of both under- and overnutrition in adolescents and to explore sex differences.

**Design:**

Analysis of anthropometric, household and neighbourhood SEP data from the Birth to Twenty Plus cohort born in 1990. Anthropometric outcomes were BMI (thinness, overweight and obesity) and percentage body fat (%BF; low, high). Associations between these and the household wealth index, caregiver education and neighbourhood SEP tertile measures were examined using binary logistic regression.

**Setting:**

Johannesburg–Soweto, South Africa.

**Subjects:**

Adolescents aged 17–19 years (*n* 2019; 48·2 % men).

**Results:**

Women had a significantly higher combined prevalence of overweight/obesity (26·2 %) than men (8·2 %) whereas men had a significantly higher prevalence of thinness than women (22·2 % *v*. 10·6 %, respectively). Having a low neighbourhood social support index was associated with higher odds of high %BF in women (OR=1·59; 95 % CI 1·03, 2·44). A low household wealth index was associated with lower odds of both overweight (OR=0·31; 95 % CI 0·12, 0·76) and high %BF in men (OR=0·28; 95 % CI 0·10, 0·78). A low or middle household wealth index was associated with higher odds of being thin in men (OR=1·90; 95 % CI 1·09, 3·31 and OR=1·80; 95 % CI 1·03, 3·15, respectively). For women, a low household wealth index was associated with lower odds of being thin (OR=0·49; 95 % CI 0·25, 0·96).

**Conclusions:**

The study highlights that even within a relatively small urban area the nutrition transition manifests itself differently in men and women and across SEP indicators. Understanding the challenges for different sexes at different ages is vital in helping to plan public health services.

Many low- and middle-income countries (LMIC) are undergoing epidemiological and health transitions with rapid increases in the incidence of overweight, obesity and diet-related chronic diseases^(^ ^1^ ^–^ ^4^ ^)^. This is particularly apparent in urban areas and is reflected by a Westernised lifestyle^(^ ^5^ ^–^ ^7^ ^)^. Twenty out of forty countries in sub-Saharan Africa are at an early stage of the nutrition transition, while several countries such as Ghana, Gabon, South Africa and Cape Verde have reached a stage where lifestyle changes contribute to poor health outcomes for a considerable part of the population^(^ ^8^ ^)^. Patterns of overweight and obesity differ between sexes in LMIC, with women generally having a higher prevalence^(^ ^9^ ^)^. Studies among women of reproductive age in LMIC have shown a positive socio-economic gradient in overweight, indicating that wealthier women experience a higher prevalence of overweight than their poorer counterparts^(^ ^10^ ^–^ ^13^ ^)^. The limited evidence on men in LMIC has revealed a similar pattern^(^ ^14^ ^,^ ^15^ ^)^. It has been observed that the burden of overweight shifts from high to low socio-economic groups as the gross domestic product of a country improves^(^ ^12^ ^–^ ^14^ ^,^ ^16^ ^,^ ^17^ ^)^. Limited evidence suggests that the transition differs between sexes, with overweight in women shifting from wealthy to poor groups at an earlier point of economic development compared with men^(^ ^14^ ^,^ ^16^ ^)^. Dinsa *et al*.^(^ ^16^ ^)^ found that children and adolescents displayed a positive relationship between socio-economic position (SEP) and obesity in both low-income and middle-income countries.

South Africa is undergoing the nutrition transition as evidenced by changes in lifestyle behaviours and the increasing prevalence of overweight and obesity^(^ ^8^ ^,^ ^18^ ^–^ ^21^ ^)^. Recent data show that South Africa has one of the highest prevalences of overweight and obesity in sub-Saharan Africa, with 30·7 % of men and 64·0 % of women being overweight or obese^(^ ^22^ ^,^ ^23^ ^)^. Paradoxically, underweight remains prevalent in the South African population affecting 12·8 % of men and 4·2 % of women^(^ ^22^ ^)^. The concurrence of under- and overnutrition at the population/neighbourhood, household or individual level is called the ‘double burden of malnutrition’^(^ ^24^ ^)^. This burden is mainly observed in LMIC^(^ ^25^ ^–^ ^27^ ^)^. Corsi *et al*.^(^ ^28^ ^)^ assessed the double burden of malnutrition in LMIC, in men and women separately, using a multilevel analysis. They showed that South Africa meets the criteria for a dual burden of malnutrition at the national level. This burden is also observed in adolescents, with a high prevalence of overweight and obesity among girls and a high prevalence of underweight in boys^(^ ^22^ ^,^ ^29^ ^)^.

It is essential to identify the factors that influence poor nutritional status in adolescents as risk factors for chronic diseases in adolescence may continue through to later life. It is also important to study neighbourhood effects in adolescents separately from adults and children because adolescence is the point in the life course when individuals become more influenced by their environment^(^ ^30^ ^)^. In high-income countries a number of frameworks have been developed to explain the importance of ecological influences on nutritional outcomes^(^ ^31^ ^–^ ^35^ ^)^. These frameworks identify the proximal determinants (individual factors such as dietary intake and physical activity behaviours, age, gender, etc.), the distal determinants which include the interpersonal (peers, neighbours and family interactions, etc.), organisational (school, church, etc.) and neighbourhood (socio-economic environment, built environment, food environment, etc.) levels, as well as the fundamental determinants which include societal and supranational levels. Despite growing evidence of the importance of an ecological approach to understanding nutritional outcomes in high-income countries, there has been little research using this approach to study adolescent nutrition in LMIC. Previous research conducted in South African adolescents aged 16 years focused on the relationship between household/neighbourhood SEP and anthropometric outcomes such as BMI, overweight/obesity, fat mass and lean mass^(^ ^36^ ^)^. However, the prevalence of underweight was not investigated and thus the dual burden of malnutrition was not studied.

The aim of the present study was to use an ecological approach to investigate the associations of household and neighbourhood SEP with indicators of both under- and overnutrition in a sample of urban South African adolescents aged 18 years. Furthermore, we aimed to explore sex differences in under- and overnutrition, after controlling for other potentially confounding variables. The present study is the first one to use a quantitative tool to measure self-perceived deprivation at the neighbourhood level among adolescents in South Africa. It is also the first cohort of adolescents that have grown up in the post-Apartheid era. Therefore it is important to understand household and neighbourhood SEP influences on the changing nutritional status of these adolescents as they enter adulthood in this rapidly changing urban environment.

## Methods

### Study design and participants

The present study was conducted in Johannesburg–Soweto, the largest urban area in South Africa. South Africa is an upper-middle-income country^(^ ^37^ ^)^ in which extremes of wealth and poverty exist. With a Gini index of 65·0, South Africa is recognised as being the most unequal society in the world^(^ ^38^ ^)^. The Gini index represents a measure of inequality in the distribution of income among individuals or households, and is measured from 0 to 100 (a Gini index of 0 means perfect equality while an index of 100 represents perfect inequality). Extreme inequalities exist within the population in the City of Johannesburg^(^ ^39^ ^)^, with high rates of poverty, unemployment, violence and crime^(^ ^40^ ^)^. The sample of adolescents comes from the Birth to Twenty Plus (Bt20+) cohort study, which has been described in detail elsewhere^(^ ^41^ ^)^. Bt20+ is a longitudinal cohort study (*n* 3273) of births that occurred in April–June 1990 to mothers who were permanent residents of Johannesburg–Soweto. The attrition rate in the study was estimated to be about 30 % when participants were 16 years old^(^ ^41^ ^)^ and remained fairly unchanged at approximately 34 % when participants were 18 years old (SA Norris, personal communication, December 2014).

### Ethical approval

The study was conducted according to the guidelines laid down in the Declaration of Helsinki and all procedures involving human subjects were approved by the ethics committees of the University of the Witwatersrand, South Africa (protocol number M980810) for primary data collection and Loughborough University, UK (generic protocol G08P9) for secondary data analyses. The primary caregiver gave written informed consent for his/her child to participate in the study and the adolescent provided written assent or consent if aged 18 years or more.

### Measurements, derived variables and data management

#### Neighbourhood- and household-level measures of socio-economic position

Members of the cohort completed an interviewer-administered questionnaire on neighbourhood and household SEP. Smoking status data and place of residence were also collected.

Neighbourhood SEP was assessed using a novel quantitative questionnaire specific to the urban South African context, developed based on qualitative work conducted with the cohort when the participants were aged 15 years^(^ ^42^ ^)^. A measure of neighbourhood SEP was built from questions related to the economic (perceptions of neighbourhood level of wealth, type of housing, facilities, neighbourhood problems, etc.) and social environments (social support, happiness, community spirit, trust, etc.) within their neighbourhoods. Neighbourhood was defined for each individual as an area that is approximately 2 km from the participant’s house in every direction. This radius was chosen as it is the distance from the residence that can be walked in approximately 20 min^(^ ^42^ ^)^. The definition of the neighbourhood was based on adolescents’ perspectives of their environment and considered both the geographical area and social networks within that specific area. Neighbourhood SEP indices were created using principal component analysis applied to proxy indicators of the neighbourhood environment in order to avoid problems of collinearity, as there were over 100 questions assessing different aspects of the neighbourhood environment in the questionnaire. This was built on indices developed by Griffiths *et al*.^(^ ^43^ ^)^ (Table 1). The neighbourhood economic index, availability of services index and problem index reflect the economic aspects of the neighbourhoods. The neighbourhood social support index reflects the social aspects of the neighbourhoods. For each index, the first component score was extracted and the assumption that all eigenvalues should be >1 was verified. Tertiles were then created for each index. The first tertile of each index represents the most disadvantaged neighbourhoods.

**Table 1:** Description of neighbourhood socio-economic environment indices created for urban South African adolescents aged 18 years

Neighbourhood economic variables: perceptions of neighbourhood wealth; outsiders’ perceptions of neighbourhood wealth; perception of equity of neighbourhood living standards; housing quality and condition; availability of yard space; parking space and fencing/walls around properties
Neighbourhood availability of services/facilities variables: primary and secondary schools; hospitals, health centres; community centres; sports facilities; parks; street lighting; piped water; policing
Neighbourhood problem variables: traffic congestion; road safety; sewerage; illegal dumping; pollution; overcrowding; in-migration of non-South Africans; homelessness; repossession of properties; unemployment; prostitution; alcohol/drug abuse; ‘shebeens’ (bars); gangs
Neighbourhood social support/happiness variables: information on the liveliness, spirit and trust levels in the neighbourhood; whether neighbours help in a time of need; whether neighbours could be trusted to look after their house; happiness and level of pride in the neighbourhood

Caregivers defined household SEP via an interviewer-administered questionnaire when adolescents were aged 16 years. A household wealth index was created using principal component analysis applied to proxy indicators of the household environment (water/toilet facilities and ownership of consumer durables) and tertiles of the household wealth index were then created. Caregiver’s education was self-reported when the adolescents were less than 18 years old and reported by the adolescents when aged 18 years or more. A categorical variable was created for caregiver education using the information given (less or equal to primary school, secondary school, higher education).

The smoking status of adolescents was assessed using a self-report questionnaire. Participants were asked whether or not they smoke and, if so, the frequency of consumption. Adolescents’ smoking status was classified as current smoker, previous smoker or never smoked. This was included because of the known association between smoking, body weight and body composition^(^ ^44^ ^)^.

A dichotomous variable was created for place of residence, representing adolescents living in Soweto or in the rest of Johannesburg metropolitan municipality. This was done using geographical mapping systems via Google (https://maps.google.co.za/) and a South African postal code system (http://postalcodez.co.za).

#### Anthropometric and body composition measures

Birth weight and weight/height at 18 years were collected following standard procedures^(^ ^45^ ^)^ by trained fieldworkers. Weight was measured to the nearest 0·1 kg using digital electronic scales (Dismed, USA). Height was measured using a Holtain stadiometer (Holtain Ltd, UK) graduated to the nearest 0·1 cm. Dual-energy X-ray absorptiometry scans were performed according to standard procedures (Hologic QDR 45000A software version 12·5:7; Hologic Inc., USA) by a trained technician. Whole body fat (kg) and percentage body fat (%BF) were calculated.

BMI was calculated as weight/height^2^ (kg/m^2^). Age- and sex-specific international cut-off points were used to define nutritional status based on BMI (thinness, normal weight, overweight and obese) for adolescents aged less than 18 years^(^ ^46^ ^,^ ^47^ ^)^. Adult cut-offs were used for adolescents aged 18 years or above^(^ ^48^ ^)^. Dichotomous variables were created for thinness (thin *v*. normal) and overweight, including obesity, because the prevalence of obesity was low (overweight/obese *v*. normal).

The ratio of overweight to thinness was calculated for men and women to indicate the coexistence of thinness and overweight at the population level. This can then be used as an indicator of the nutrition transition^(^ ^1^ ^,^ ^2^ ^)^. Age- and sex-specific cut-off points based on the US Third National Health and Nutrition Examination Survey (NHANES III) were used to define the different classes of fat status according to %BF (below average, ≤15·00th percentile; normal, ≥15·01th and ≤85·00th percentile; above average, ≥85·01th percentile)^(^ ^49^ ^)^. Dichotomous variables were created for high %BF (high *v*. normal) and low %BF (low *v*. normal). Low birth weight was defined as weight at birth<2500 g^(^ ^50^ ^)^.

#### Intrinsic measures

Data on age, sex, pubertal development and population group were collected. The pubertal stage of development was assessed using a self-completed questionnaire, which uses the Tanner scaling of pubic hair and breast/genitalia development^(^ ^51^ ^)^. This tool has been validated in black South African adolescents aged 10–18 years^(^ ^52^ ^)^. Women were also asked to specify whether or not they had achieved menarche and if so, the date was recorded. Population group of the child was identified by the mother as black African, white, ‘coloured’ and Indian (terms used in the Bt20+ questionnaire) at birth. The term ‘mixed ancestry’ is used to describe the ‘coloured’ group in the present paper.

As menarche was a more readily defined event in pubertal development, age of menarche was used to indicate early and late sexual development in women. The Tanner stages were used in men as a proxy of pubertal development. Binary variables were created for pubertal development (for men: Tanner stage 2–3 (late maturers) *v*. stage 4–5 (early maturers); for women: age of entry into menarche, <13 years *v*. ≥13 years). Stage of pubertal development was included to control for the known changes in body composition during different stages of adolescence^(^ ^29^ ^)^.

### Statistical analysis

Participants with data on population group, sex, pubertal development, smoking status, birth weight, SEP and anthropometry were included. Descriptive statistics on sociodemographic factors were performed for all adolescents with a valid BMI (maximum sample size *n* 2019). Descriptive statistics on anthropometric factors were completed on the maximum sample size for each outcome (*n* 2019 for BMI and *n* 1728 for %BF). Due to their small sample size (*n* 27, 1·3 %), Indian/Asian participants were only included in the descriptive analysis. Analyses were stratified by sex due to different nutritional status patterns between men and women. Univariate analyses were performed on the maximum sample size for each outcome. The multivariate analysis was based on the sample that had complete data for all variables included in this stage of analysis.

Associations between intrinsic variables, household and neighbourhood SEP tertile measures and anthropometric outcomes were examined using binary logistic regression. Univariate models were generated, followed by stepwise multivariate regression analyses. The variables shown in the fully adjusted models were retained based on their significance in the univariate models (*P*<0·1).

Variables were entered in the following order: intrinsic-level variables (age, age of entry into menarche, Tanner stage of pubertal development, population group, low birth weight) followed by the neighbourhood-level variables (neighbourhood SEP, place of residence) and finally the household-level variables (caregiver education, household wealth index, smoking status). This model-building process allowed for the assessment of the mediating effect of the household-level variables on the association between neighbourhood SEP variables and the different anthropometric outcomes. All models using %BF were adjusted for height^(^ ^53^ ^)^.

Analyses were conducted using the statistical software package Stata/SE version 12 (2011). The type I error risk was set at 0·05. Results are presented as odds ratios and 95 % confidence intervals.

## Results

The sample was composed mainly of black adolescents (81·1 %), with 10·8 % mixed ancestry, 6·7 % white and 1·3 % Indian/Asian (Table 2). Eighty-two per cent of the sample lived in Soweto. In terms of pubertal development, 86 % of men had achieved Tanner stage 4 or 5 at 18 years. The mean age of entry into menarche was 12·7 years. Of the adolescents’ caregivers, 71·4 % had achieved a secondary school education, with no significant differences between sexes. The proportion of current smokers was significantly higher in men than women (45·2 % *v*. 23·2 %, respectively, *P*<0·0001). The distribution of people in each tertile of the household and neighbourhood SEP indices was similar between genders apart from the neighbourhood social support index, where the proportion of people in the first tertile (unfavourable social environment) was higher in women than in men because of the small range of scores generated from the principal component analysis, which limited the designation of the sample into even tertiles.

**Table 2:** Sociodemographic characteristics of the sample: adolescents aged 17–19 years, Johannesburg–Soweto, South Africa, Birth to Twenty Plus (Bt20+) cohort

	Total group (*n* 2019)		Men (*n* 974)		Women (*n* 1045)		
Variable	Mean	sd	Mean	sd	Mean	sd	Test
Intrinsic factors							
Age (years)	18·21	0·57	18·24	0·58	18·18	0·57	*P*=0·03*†
Age of entry into menarche (years)	–	–	–	–	12·70	1·25	–
Birth weight (kg)	3·11	0·51	3·11	0·52	3·01	0·49	*P*<0·0001***†
	*n*	%	*n*	%	*n*	%	
Population group							
White	136	6·7	65	6·7	71	6·8	*P*=0·85‡
Black African	1638	81·1	792	81·3	846	80·9	
Mixed ancestry	218	10·8	102	10·5	116	11·1	
Indian/Asian	27	1·3	15	1·5	12	1·2	
Physical maturation (Tanner stages)							
Early stages (2–3)	–	–	111	14·1	–	–	–
Later stages (4–5)	–	–	676	85·9	–	–	
Age of entry into menarche							
9–13 (years)	–	–	–	–	397	43·1	–
13–17 (years)	–	–	–	–	523	56·8	
Low birth weight (<2500 g)							
No	1674	89·0	818	90·2	856	87·9	*P*=0·11‡
Yes	207	11·0	89	9·8	118	12·1	
Household socio-economic factors							
Caregiver education							
Less than or primary school	265	14·6	131	14·9	134	14·3	*P*=0·78‡
Secondary school	1296	71·4	629	71·6	667	71·2	
Higher education	254	14·0	118	13·5	136	14·5	
Adolescent smoking status							
Never smoked	485	32·0	176	24·6	309	38·6	*P*<0·0001***‡
Previous smoker	521	34·4	215	30·1	306	38·2	
Current smoker	509	33·6	323	45·2	186	23·2	
Household wealth index							
1st tertile	612	34·3	305	35·4	307	33·3	*P*=0·41‡
2nd tertile	612	34·3	299	34·7	313	34·0	
3rd tertile	558	31·4	257	29·9	301	32·7	
Neighbourhood socio-economic factors							
Place of residence							
Soweto	1338	82·1	664	84·4	674	80·0	*P*=0·02*‡
Metropolitan Johannesburg	291	17·9	123	15·6	168	20·0	
Neighbourhood economic index							
1st tertile	663	35·2	293	32·5	370	37·7	*P*=0·06^(^*^)^ ‡
2nd tertile	590	31·3	296	32·9	294	29·9	
3rd tertile (high)	630	33·5	312	34·6	318	32·4	
Neighbourhood availability of services index							
1st tertile	627	33·4	297	33·1	330	33·6	*P*=0·50‡
2nd tertile	641	34·1	297	33·1	344	35·0	
3rd tertile (high)	611	32·5	303	33·8	308	31·4	
Neighbourhood problem index							
1st tertile	623	33·5	307	34·3	316	32·6	*P*=0·22‡
2nd tertile	617	33·1	306	34·2	311	32·1	
3rd tertile (low)	622	33·4	281	31·5	341	35·3	
Neighbourhood social support index							
1st tertile	622	33·1	270	29·9	352	35·9	*P*=0·02*‡
2nd tertile	629	33·4	308	34·2	321	32·8	
3rd tertile (favourable)	631	33·5	324	35·9	307	31·3	

^(^*^)^ *P*<0·10, *P<0·05, **P<0·01, ***P<0·001.

† *P* value from *t* test.

‡ *P* value from *χ* ^2^ test.

BMI was significantly greater in women (22·0 *v*. 19·9 kg/m^2^, *P*<0·0001; Table 3). The prevalence of thinness at 18 years was 22·2 % in men, as opposed to 10·6 % in women (*P*<0·0001). The proportion of overweight was approximately three times higher in women than in men (17·9 % *v*. 6·1 %, *P*<0·0001), with a similar pattern observed for obesity (8·3 % *v*. 2·2 %, *P*<0·0001). The overweight:thinness ratio was 0·37 in men as opposed to 2·47 in women.

**Table 3:** Anthropometrics characteristics of the sample: adolescents aged 17–19 years, Johannesburg–Soweto, South Africa, Birth to Twenty Plus (Bt20+) cohort

	Total group (*n* 2019)		Men (*n* 974)		Women (*n* 1045)		
Variable	Mean or median	sd or IQR	Mean or median	sd or IQR	Mean or median	sd or IQR	Test
Height (cm)	165·5	8·8	171·5	6·9	159·9	6·4	*P*<0·0001***§
Weight (kg)	57·6	51·8–64·8	58·3	53·6–65·3	56·5	50·2–64·2	*P*<0·0001***||
BMI (kg/m^2^)	20·8	19·0–23·5	19·9	18·5–21·7	22·0	19·8–25·1	*P*<0·0001***||
	*n*	%	*n*	%	*n*	%	
BMI in four classes†							
Thinness	327	16·2	216	22·2	111	10·6	*P*<0·0001***¶
Normal	1338	66·3	678	69·6	660	63·2	
Overweight	245	12·2	59	6·1	187	17·9	
Obese	108	5·3	21	2·2	87	8·3	
Overweight (including obesity)	354	17·5	80	8·2	274	26·2	*P*<0·0001***¶
Overweight:thinness ratio	1·1		0·4		2·5	
	Total group (*n* 1728)		Men (*n* 839)		Women (*n* 889)		
Variable	Mean	sd	Median	IQR	Mean	sd	Test
%BF	23·8	11·7	12·05	9·9–15·3	33·2	7·11	*P*<0·0001***||
	*n*	%	*n*	%	*n*	%	
%BF in three classes‡							
Low %BF (≤15·00th percentile)	626	36·2	501	59·8	125	14·0	*P*<0·0001***¶
Normal %BF (≥15·01 and ≤85·00th percentile)	845	48·9	294	35·0	551	62·0	*P*<0·0001***¶
High %BF (≥85·01th percentile)	257	14·9	44	5·2	213	24·0	*P*<0·0001***¶

IQR, interquartile range; %BF, percentage body fat. ^(^*^)^ *P*<0·10, *P<0·05, **P<0·01, ***P<0·001.

† Categories of BMI were defined using age- and sex-specific international cut-offs for BMI for <18 years^(^ ^46^ ^,^ ^47^ ^)^ and ≥18 years^(^ ^48^ ^)^.

‡ Categories of %BF were defined using age- and sex-specific cut-off points based on the US Third National Health and Nutrition Examination Survey^(^ ^49^ ^)^.

§ *P* value from *t* test.

|| *P* value from Mann–Whitney test.

¶ *P* value from *χ* ^2^ test.

In men, 5·2 % had a high %BF and 59·8 % had a low %BF. In women, 24·0 % had a high %BF and 14·0 % had a low %BF.

Table 4 shows the predictors of overweight and high %BF from univariate analyses for men and women separately. In men, significant predictors of overweight were population group, caregiver education and household wealth index. Predictors of high %BF in men were population group and household wealth index. In women, late entry into menarche was negatively associated with overweight and high %BF. Furthermore, having a low neighbourhood social support index was associated with higher odds of high %BF in women.

**Table 4:** Predictors of overweight and high %BF from univariate logistic regression analyses for 18-year-old men and women, Johannesburg–Soweto, South Africa, Birth to Twenty Plus (Bt20+) cohort

	Overweight† (men)				Overweight† (women)				High %BF‡ (men)				High BF‡ (women)			
	*n*	%	OR	95 % CI	*n*	%	OR	95 % CI	*n*	%	OR	95 % CI	*n*	%	OR	95 % CI
Intrinsic factors																
Age (years)	758		*P*=0·201		934		*P*=0·30		338		*P*=0·69		764		*P*=0·19	
17–18 years§	267	8·6	1·00	–	385	31·2	1·00	–	140	12·1	1·00	–	355	30·1	1·00	–
≥18 years	491	11·6	1·39	0·84, 2·32	549	28·0	0·86	0·65, 1·14	198	13·6	1·14	0·60, 2·18	409	25·9	0·81	0·59, 1·11
Population group	750		*P*=0·001**		924		*P*=0·14		329		*P*=0·001**		757		*P*=0·16	
Black African§	620	8·4	1·00	–	774	30·4	1·00	–	281	11·0	1·00	–	640	14·6	1·00	–
White	63	22·2	3·12	1·61, 6·03	61	31·1	1·04	0·59, 1·82	23	0·0	–	–	41	28·7	0·42	0·17, 1·03
Mixed ancestry	67	16·4	2·14	1·06, 4·35	89	20·2	0·58	0·34, 0·99	25	36·0	4·54	1·85, 11·1	76	26·3	0·88	0·52, 1·52
Low birth weight (<2500 g)	706		*P*=0·08 ^(^*^)^		869		*P*=0·17		336		*P*=0·52		761		*P*=0·27	
No§	649	11·4	1·00	–	769	30·7	1·00	–	313	13·4	1·00	–	670	29·7	1·00	–
Yes	57	3·5	0·28	0·07, 1·18	100	24·0	0·71	0·44, 1·16	23	8·7	0·61	0·14, 2·72	91	23·1	0·75	0·44, 1·25
Pubertal development	622		*P*=0·489		825		*P*<0·0001***		308		*P*=0·36		732		*P*=0·001**	
Early stage§,||	87	8·1	1·00	–	367	36·5	1·00	–	38	15·8	1·00	–	325	34·5	1·00	–
Late stage¶	535	10·5	1·34	0·59, 3·03	458	24·5	0·56	0·42, 0·76	270	10·7	0·64	0·25, 1·66	407	23·3	0·58	0·42, 0·80
Household socio-economic factors																
Caregiver education	683		*P*=0·001**		837		*P*=0·67		795		*P*=0·37		678		*P*=0·24	
≤Primary school	97	8·3	0·32	0·13, 0·77	119	29·4	1·22	0·69, 2·16	120	24·2	1·20	0·63, 2·27	90	10	0·49	0·21, 1·13
Secondary school	490	9·0	0·35	0·20, 0·62	596	29·4	1·22	0·78, 1·90	575	27·3	1·41	0·84, 2·36	480	14·2	0·73	0·42, 1·26
Higher education§	96	21·9	1·00	–	122	25·4	1·00	–	100	21·0	1·00	–	108	18·5	1·00	–
Household wealth index	675		*P*=0·0002***		821		*P*=0·25		297		*P*=0·007**		674		*P*=0·22	
1st tertile	230	6·1	0·30	0·16, 0·58	285	25·6	0·77	0·53, 1·12	96	6·2	0·24	0·09, 0·62	224	24·1	0·78	0·51, 1·18
2nd tertile	223	8·1	0·41	0·23, 0·75	274	31·4	1·02	0·71, 1·47	105	11·4	0·46	0·21, 0·99	233	31·3	1·11	0·74, 1·67
3rd tertile (high)§	222	17·6	1·00	–	262	30·9	1·00	–	96	21·9	1·00	–	217	29·0	1·00	–
Adolescent smoking status	558		*P*=0·435		715		*P*=0·44		271		*P*=0·19		633		*P*=0·67	
Never smoked§	138	11·6	1·00	–	278	32·4	1·00	–	77	11·7	1·00	–	242	27·3	1·00	–
Previous smoker	173	13·3	1·17	0·59, 2·31	270	27·4	0·79	0·55, 1·14	92	18·5	1·71	0·72, 4·09	245	30·6	1·17	0·79, 1·74
Current smoker	247	9·3	0·78	0·40, 1·54	167	30·5	0·92	0·61, 1·39	102	9·8	0·82	0·32, 2·13	146	27·4	1·00	0·63, 1·59
Neighbourhood socio-economic factors																
Neighbourhood economic index	709		*P*=0·085^(^*^)^		878		*P*=0·29		309		*P*=0·65		718		*P*=0·54	
1st tertile	233	10·7	0·77	0·40, 1·34	331	31·4	1·01	0·72, 1·42	102	12·8	1·13	0·49, 2·61	284	28·9	0·98	0·67, 1·44
2nd tertile	224	7·1	0·49	0·26, 0·92	265	26·0	0·78	0·53, 1·13	102	15·7	1·44	0·64, 3·22	201	25·0	0·80	0·52, 1·23
3rd tertile (high)§	252	13·5	1·00	–	282	31·2	1·00	–	105	11·4	1·00	–	233	29·2	1·00	–
Neighbourhood availability of services index	706		*P*=0·34		878		*P*=0·50		306		*P*=0·92		718		*P*=0·75	
1st tertile	228	8·3	0·74	0·40, 1·37	300	32·0	1·19	0·83, 1·71	95	13·7	1·16	0·51, 2·64	254	27·9	0·96	0·65, 1·44
2nd tertile	231	12·5	1·17	0·67, 2·04	309	28·2	0·99	0·69, 1·43	103	13·6	1·15	0·51, 2·58	241	25·7	0·86	0·57, 1·29
3rd tertile (high)§	247	10·9	1·00	–	269	28·3	1·00	–	108	12·0	1·00	–	223	28·7	1·00	–
Neighbourhood problem index	704		*P*=0·082^(^*^)^		868		*P*=0·93		304		*P*=0·97		709		*P*=0·86	
1st tertile	228	7·9	0·52	0·28, 0·95	287	30·0	1·07	0·75, 1·53	99	14·1	1·08	0·49, 2·40	241	27·8	1·07	0·71, 1·60
2nd tertile	244	9·8	0·66	0·37, 1·15	280	29·3	1·03	0·72, 1·48	99	13·1	0·99	0·44, 2·23	230	28·7	1·12	0·74, 1·68
3rd tertile (low)§	232	14·2	1·00	–	301	28·6	1·00	–	106	13·2	1·00	–	238	26·5	1·00	–
Neighbourhood social support index	710		*P*=0·99		877		*P*=0·69		311		*P*=0·17		718		*P*=0·046*	
1st tertile	217	10·6	0·99	0·55, 1·79	321	28·0	0·86	0·60, 1·22	97	18·6	2·05	0·91, 4·59	265	27·2	1·26	0·84, 1·91
2nd tertile	249	10·8	1·02	0·58, 1·80	287	30·0	0·94	0·66, 1·35	104	11·5	1·17	0·49, 2·79	229	33·2	1·68	1·11, 2·55
3rd tertile (favourable)§	244	10·7	1·00	–	269	31·2	1·00	–	110	10·0	1·00	–	224	22·8	1·00	–
Place of residence	623		*P*=0·031*		749		*P*=0·80		271		*P*=0·18		620		*P*=0·61	
Soweto§	518	9·1	1·00	–	606	29·0	1·00	–	228	14·9	1·00	–	505	27·5	1·00	–
Metropolitan Johannesburg	105	16·2	1·94	1·06, 3·52	143	28·0	0·95	0·63, 1·42	43	7·0	0·43	0·12, 1·46	115	25·2	0·89	0·56, 1·41

%BF, percentage body fat. ^(^*^)^ *P*<0·10, *P<0·05, **P<0·01, ***P<0·001.

† Overweight was defined using age- and sex-specific international cut-offs for BMI for <18 years^(^ ^46^ ^)^ and ≥18 years^(^ ^48^ ^)^.

‡ High %BF was defined as fat mass ≥85·01th percentile^(^ ^49^ ^)^.

§ Reference category.

|| In men, early stage of pubertal development refers to Tanner stages 2–3 (late maturers). In women, it refers to age of entry into menarche <13 years (early maturers).

¶ In men, late stage of pubertal development refers to Tanner stages 4–5 (early maturers). In women, it refers to age of entry into menarche ≥13 years (late maturers).

Overweight for women was not examined in multivariate analyses because no univariate association with household or neighbourhood SEP was found. Table 5 shows the stepwise multivariate analysis for the predictors of overweight in men. In the fully adjusted model, the variables remaining significant were the secondary school education level of caregiver and the first tertile of the household wealth index. Attaining a secondary school educational level compared with a higher level (OR=0·39; 95 % CI 0·17, 0·88) and being in the lowest tertile of the household wealth index compared with the highest tertile (OR=0·31; 95 % CI 0·12, 0·76) decreased the odds of being overweight in men. The neighbourhood economic index became significantly associated with overweight in the fully adjusted model. Being in the first tertile of the neighbourhood economic index (least wealthy) increased the odds for being overweight (OR=3·00; 95 % CI 1·25, 7·20).

**Table 5:** Odds ratios and 95 % confidence intervals for overweight† from the adjusted logistic regression analyses in 18-year-old men (*n* 475), Johannesburg–Soweto, South Africa, Birth to Twenty Plus (Bt20+) cohort

		Step 1		Step 2		Step 3	
	*n*	Adjusted OR‡	95 % CI	Adjusted OR‡	95 % CI	Adjusted OR‡	95 % CI
Intrinsic factors							
Population group (black African§)	396						
White	37	3·59**	1·61, 8·04	3·97^(^*^)^	0·98, 16·1	1·98	0·48, 8·24
Mixed ancestry	42	1·68	0·66, 4·27	1·67	0·63, 4·44	1·25	0·45, 3·46
Low birth weight (<2500 g) (no§)	438						
Yes	37	0·48	0·11, 2·08	0·49	0·11, 2·14	0·46	0·10, 2·06
Neighbourhood socio-economic factors							
Neighbourhood economic index (3rd tertile (high)§)	156						
1st tertile	157			1·89	0·85, 4·18	3·00*	1·25, 7·20
2nd tertile	162			0·93	0·40, 2·20	1·23	0·50, 3·02
Neighbourhood problem index (3rd tertile (low)§)	160						
1st tertile	156			0·63	0·28, 1·39	0·75	0·33, 1·70
2nd tertile	159			0·78	0·38, 1·60	0·87	0·41, 1·82
Place of residence (Soweto§)	399						
Metropolitan Johannesburg	76			0·94	0·30, 2·93	0·77	0·24, 2·47
Household socio-economic factors							
Caregiver education (higher education§)	70						
≤Primary school	69					0·50	0·15, 1·63
Secondary school	336					0·39*	0·17, 0·88
Household wealth index (3rd tertile (high)§)	144						
1st tertile	176					0·31*	0·12, 0·76
2nd tertile	155					0·45^(^*^)^	0·20, 1·02
Cox/Snell and Nagelkerke *R* ^2^ estimates		0·022, 0·043		0·032, 0·064		0·061, 0·122	
Deviance		317·74		312·78		298·40	

Results are presented only for men in this table as pubertal development was the only factor significant for women in univariate analysis, thus no model could be built in multivariate analysis. ^(^*^)^ *P*<0·10, *P<0·05, **P<0·01, ***P<0·001.

† Overweight was defined using age- and sex-specific international cut-offs for BMI for <18 years^(^ ^46^ ^)^ and ≥18 years^(^ ^48^ ^)^.

‡ OR adjusted by logistic regression.

§ Reference category.

Table 6 shows the stepwise multivariate analysis for the predictors of high %BF in men and women. In men, the fully adjusted model showed that adolescents in the lowest tertile of the household wealth index had significantly reduced odds of having high %BF (OR=0·28; 95 % CI 0·10, 0·78) compared with adolescents in the highest tertile. Mixed ancestry adolescents had significantly higher odds of having high %BF (OR=3·49; 95 % CI 1·29, 9·43). In women, in the fully adjusted model both early menarche and being in the second tertile of the neighbourhood social support index remained significant predictors of having high %BF, with similar associations to those observed in the univariate analysis (OR=1·59; 95 % CI 1·03, 2·44 for the second tertile *v*. the third tertile of the index (favourable social environment)).

**Table 6:** Odds ratios and 95 % confidence intervals for high %BF† from the adjusted logistic regression analyses in 18-year-old men and women, Johannesburg–Soweto, South Africa, Birth to Twenty Plus (Bt20+) cohort

		Step 1		Step 2	
	*n*	Adjusted OR‡	95 % CI	Adjusted OR‡	95 % CI
Men (*n* 290)					
Intrinsic factors					
Height	290	1·03	0·97, 1·09	1·02	0·96, 1·08
Population group (black African§)	249				
White	18				
Mixed ancestry	23	5·13***	2·02, 13·0	3·49*	1·29, 9·43
Household socio-economic factors					
Household wealth index (3rd tertile (high)§)	90				
1st tertile	96			0·28*	0·10, 0·78
2nd tertile	104			0·52	0·22, 1·21
Cox/Snell and Nagelkerke *R* ^2^ estimates		0·043, 0·080		0·065, 0·121	
Deviance		200·6		194·2	
Women (*n* 692)					
Intrinsic factors					
Height	692	0·96**	0·93, 0·99	0·96**	0·93, 0·99
Age of entry into menarche (<13 years§)	309				
≥13 years	383	0·64**	0·45, 0·89	0·65**	0·46, 0·92
Neighbourhood socio-economic factors					
Neighbourhood social support index (3rd tertile (favourable)§)	213				
1st tertile	259			1·20	0·79, 1·84
2nd tertile	220			1·59*	1·03, 2·44
Cox/Snell and Nagelkerke *R* ^2^ estimates		0·024, 0·035		0·031, 0·044	
Deviance		799·70		795·03	

%BF, percentage body fat. ^(^*^)^ *P*<0·10, *P<0·05, **P<0·01, ***P<0·001.

† High %BF was defined as fat mass ≥85·01th percentile^(^ ^49^ ^)^.

‡ OR adjusted by logistic regression.

§ Reference category.

Table 7 shows the predictors of thinness and low %BF from univariate analyses for men and women. In men, the significant predictors of thinness were population group, low birth weight and household wealth index and the ones for low %BF were age, population group, low birth weight, caregiver education and smoking status. In women, the predictors of thinness were population group and household wealth index. Predictors for low %BF were age and population group. None of the neighbourhood socio-economic variables were significantly associated with thinness or low %BF in men or women.

**Table 7:** Predictors of thinness and low %BF from univariate logistic regression analyses for 18-year-old men and women, Johannesburg–Soweto, South Africa, Birth to Twenty Plus (Bt20+) cohort

	Thinness† (men)				Thinness† (women)				Low %BF‡ (men)				Low %BF‡ (women)			
	*n*	%	OR	95 % CI	*n*	%	OR	95 % CI	*n*	%	OR	95 % CI	*n*	%	OR	95 % CI
Intrinsic factors																
Age	894		*P*=0·48		771		*P*=0·80		795		*P*=0·004**		676		*P*=0·001**	
17–18 years§	316	22·8	1·00	–	311	14·8	1·00	–	282	56·4	1·00	–	283	12·4	1·00	–
≥18 years	578	24·9	1·12	0·81, 1·55	460	14·1	0·95	0·63, 1·43	513	66·7	1·54	1·15, 2·08	393	22·9	2·10	1·38, 3·22
Population group	882		*P*=0·0001***		761		*P*=0·0002***		787		*P*=0·014*		671		*P*=0·003**	
Black African§	740	23·2	1·00	–	611	11·8	1·00	–	661	62·2	1·00	–	543	16·0	1·00	–
White	51	3·9	0·13	0·03, 0·56	52	19·2	1·78	0·86, 3·71	52	55·8	0·77	0·43, 1·35	51	31·4	2·39	1·27, 4·52
Mixed ancestry	91	38·5	2·06	1·31, 3·25	98	27·5	2·85	1·71, 4·72	74	78·4	2·20	1·24, 3·91	77	27·3	1·96	1·13, 3·41
Low birth weight (<2500 g)	831		*P*=0·004**		714		*P*=0·19		789		*P*=0·045*		673		*P*=0·99	
No§	744	22·7	1·00	–	620	14	1·00	–	710	61·8	1·00	–	587	18·6	1·00	–
Yes	87	36·8	1·98	1·24, 3·16	94	19·1	1·45	0·83, 2·54	79	73·4	1·70	1·01, 2·87	86	18·6	1·00	0·56, 1·79
Pubertal development	724		*P*=0·94		674		*P*=0·11		703		*P*=0·44		635		*P*=0·06^(^*^)^	
Early stage§,||	104	23·1	1·00	–	263	11·4	1·00	–	91	64·8	1·00	–	247	13·8	1·00	–
Late stage¶	620	22·7	0·98	0·60, 1·61	411	15·8	1·46	0·92, 2·32	612	60·6	0·83	0·53, 1·32	388	19·6	1·53	0·98, 2·37
Household socio-economic factors																
Caregiver education	805		*P*=0·61		696		*P*=0·93		716		*P*=0·004**		612		*P*=0·29	
≤Primary school	123	27·6	1·30	0·70, 2·42	99	15·1	1·16	0·53, 2·55	111	67·6	2·36	1·34, 4·15	89	15·7	0·59	0·28, 1·24
Secondary school	585	23·8	1·06	0·64, 1·77	492	14·4	1·09	0·59, 2·03	509	63·5	1·97	1·27, 3·05	427	17·8	0·69	0·40, 1·17
Higher education§	97	22·7	1·00	–	105	13·3	1·00	–	96	46·9	1·00	–	96	24·0	1·00	–
Household wealth index	790		*P*=0·009**		681		*P*=0·021*		701		*P*=0·39		593		*P*=0·71	
1st tertile	291	25·8	1·81	1·16, 2·84	234	9·4	0·48	0·27, 0·84	263	65·8	1·31	0·89, 1·93	204	16·7	0·81	0·48, 1·35
2nd tertile	281	27·0	1·94	1·24, 3·03	227	17·2	0·96	0·59, 1·57	253	63·2	1·17	0·79, 1·73	197	18·8	0·94	0·57, 1·55
3rd tertile (high)§	218	16·1	1·00	–	220	17·7	1·00	–	185	59·5	1·00	–	192	19·8	1·00	–
Adolescent smoking status	652		*P*=0·68		586		*P*=0·90		616		*P*=0·035*		551		*P*=0·96	
Never smoked§	160	23·8	1·00	–	219	14·2	1·00	–	159	57·2	1·00	–	215	18·1	1·00	–
Previous smoker	192	21·9	0·89	0·54, 1·48	232	15·5	1·11	0·66, 1·87	175	57·1	0·99	0·64, 1·54	208	18·3	1·01	0·61, 1·65
Current smoker	300	25·3	1·09	0·69, 1·70	135	14·1	0·99	0·54, 1·84	282	67·4	1·54	1·03, 2·30	128	17·2	0·94	0·53, 1·66
Neighbourhood socio-economic factors																
Neighbourhood economic index	826		*P*=0·47		721		*P*=0·70		731		*P*=0·94		633		*P*=0·38	
1st tertile	268	22·4	1·05	0·70, 1·57	266	14·7	0·92	0·57, 1·51	242	63·2	1·02	0·71, 1·48	239	15·5	0·72	0·44, 1·17
2nd tertile	280	25·7	1·26	0·85, 1·86	225	12·9	0·79	0·47, 1·35	240	64·2	1·07	0·74, 1·54	187	19·3	0·94	0·57, 1·54
3rd tertile (high)§	278	21·6	1·00	–	230	15·6	1·00	–	249	62·6	1·00	–	207	20·3	1·00	–
Neighbourhood availability of services index	822		*P*=0·36		723		*P*=0·43		728		*P*=0·34		636		*P*=0·72	
1st tertile	278	24·8	1·30	0·87, 1·93	234	12·8	0·73	0·43, 1·22	249	67·1	1·29	0·89, 1·86	219	16·4	0·87	0·52, 1·44
2nd tertile	268	24·6	1·28	0·86, 1·92	257	13·6	0·78	0·47, 1·28	234	62	1·03	0·71, 1·49	222	19·4	1·06	0·65, 1·73
3rd tertile (high)§	276	20·3	1·00	–	232	16·8	1·00	–	245	61·2	1·00	–	195	18·5	1·00	–
Neighbourhood problem index	819		*P*=0·09^(^*^)^		714		*P*=0·60		723		*P*=0·31		624		*P*=0·29	
1st tertile	289	27·3	1·53	1·02, 2·29	230	12·6	0·77	0·46, 1·29	251	66·1	1·32	0·91, 1·92	210	17·1	0·78	0·48, 1·28
2nd tertile	282	22·0	1·14	0·75, 1·74	229	13·5	0·84	0·51, 1·40	244	64·8	1·24	0·86, 1·80	193	15	0·67	0·40, 1·12
3rd tertile (low)§	248	19·8	1·00	–	255	15·7	1·00	–	228	59·7	1·00	–	221	20·8	1·00	–
Neighbourhood social support index	826		*P*=0·18		720		*P*=0·26		731		*P*=0·78		633		*P*=0·65	
1st tertile	247	21·5	0·74	0·50, 1·10	262	11·8	0·65	0·39, 1·09	208	62	0·99	0·68, 1·44	231	16·5	0·89	0·55, 1·47
2nd tertile	281	21·0	0·72	0·49, 1·06	235	14·5	0·82	0·50, 1·36	261	64·8	1·11	0·78, 1·59	191	19·9	1·13	0·69, 1·86
3rd tertile (favourable)§	298	26·9	1·00	–	223	17·0	1·00	–	262	62·2	1·00	–	211	18·0	1·00	–
Place of residence	723		*P*=0·13		626		*P*=0·097^(^*^)^		636		*P*=0·42		552		*P*=0·075^(^*^)^	
Soweto§	617	23·7	1·00	–	498	13·6	1·00	–	537	63·9	1·00	–	439	16·7	1·00	–
Metropolitan Johannesburg	106	17·0	0·66	0·38, 1·13	128	19·5	1·53	0·92, 2·55	99	59·6	0·83	0·54, 1·29	113	23·9	1·57	0·95, 2·59

%BF, percentage body fat. ^(^*^)^ *P*<0·10, *P<0·05, **P<0·01, ***P<0·001.

† Thinness was defined using age- and sex-specific international cut-offs for BMI for <18 years^(^ ^47^ ^)^ and ≥18 years^(^ ^48^ ^)^.

‡ Low %BF was defined as fat mass ≤15·00th percentile^(^ ^49^ ^)^.

§ Reference category.

|| In men, early stage of pubertal development refers to Tanner stages 2–3 (late maturers). In women, it refers to age of entry into menarche <13 years (early maturers).

¶ In men, late stage of pubertal development refers to Tanner stages 4–5 (early maturers). In women, it refers to age of entry into menarche ≥13 years (late maturers).

Table 8 shows the stepwise multivariate analysis for the predictors of thinness separately for men and women. In men, the final step identified mixed ancestry (OR=2·33; 95 % CI 1·33, 4·07), having low birth weight (OR=1·91; 95 % CI 1·12, 3·26) and being in the lowest (OR=1·90; 95 % CI 1·09, 3·31) or middle tertile (OR=1·80; 95 % CI 1·03, 3·15) of the household wealth index as significant predictors of thinness. In women, mixed ancestry adolescents had higher odds of thinness compared with the black group (OR=2·98; 95 % CI 1·61, 5·53). Being in the lowest tertile of the household wealth index remained a significant predictor of thinness, with those in the lowest tertile of the household wealth index displaying lower odds of thinness (OR=0·49; 95 % CI 0·25, 0·96).

**Table 8:** Odds ratios and 95 % confidence intervals for thinness† from the adjusted logistic regression analyses in 18-year-old men and women, Johannesburg–Soweto, South Africa, Birth to Twenty Plus (Bt20+) cohort

		Step 1		Step 2		Step 3	
	*n*	Adjusted OR‡	95 % CI	Adjusted OR‡	95 % CI	Adjusted OR‡	95 % CI
Men (*n* 667)							
Intrinsic factors							
Population group (black African§)	569						
White	34	0·11*	0·01, 0·83	0·12*	0·02, 0·90	0·20	0·02, 1·53
Mixed ancestry	64	2·15**	1·24, 3·71	2·17**	1·25, 3·76	2·33**	1·33, 4·07
Low birth weight (<2500 g) (no§)	593						
Yes	74	1·87*	1·10, 3·18	1·86*	1·09, 3·17	1·91*	1·12, 3·26
Neighbourhood socio-economic factors							
Neighbourhood problem index (3rd tertile (low)§)	212						
1st tertile	234			1·22	0·77, 1·93	1·12	0·71, 1·80
2nd tertile	221			1·00	0·62, 1·61	0·96	0·59, 1·55
Household socio-economic factors							
Household wealth index (3rd tertile (high)§)	169						
1st tertile	262					1·90*	1·09, 3·31
2nd tertile	236					1·80*	1·03, 3·15
Cox/Snell and Nagelkerke *R* ^2^ estimates		0·035, 0·053		0·036, 0·055		0·045, 0·069	
Deviance		682·61		681·55		675·43	
Women (*n* 551)							
Intrinsic factors							
Population group (black African§)	438						
White	34	1·49	0·59, 3·77	1·93	0·61, 6·08	1·72	0·52, 5·70
Mixed ancestry	79	3·04***	1·74, 5·30	3·33***	1·82, 6·13	2·98**	1·61, 5·53
Neighbourhood socio-economic factors							
Place of residence (Soweto§)	445						
Metropolitan Johannesburg	106			0·76	0·36, 1·57	0·68	0·33, 1·43
Household socio-economic factors							
Household wealth index (3rd tertile (high)§)	164						
1st tertile	199					0·49*	0·25, 0·96
2nd tertile	188					1·08	0·60, 1·92
Cox/Snell and Nagelkerke *R* ^2^ estimates		0·025, 0·044		0·026, 0·046		0·039, 0·068	
Deviance		459·70		459·15		451·90	

^(^*^)^ *P*<0·10, *P<0·05, **P<0·01, ***P<0·001.

† Thinness was defined using age- and sex-specific international cut-offs for BMI for <18 years^(^ ^46^ ^,^ ^47^ ^)^ and ≥18 years^(^ ^48^ ^)^.

‡ OR adjusted by logistic regression.

§ |Reference category.

Table 9 shows the stepwise multivariate analysis for the predictors of low %BF in men and women. In men, the final step shows that mixed ancestry adolescents had higher odds of having low %BF in comparison to black adolescents (OR=2·38; 95 % CI 1·20, 4·72). Adolescents aged 18 years or above compared with those aged less than 18 years had higher odds of having low %BF (OR=1·74; 95 % CI 1·20, 2·51). Current smokers had higher odds than non-smokers of having low %BF (OR=1·60; 95 % CI 1·03, 2·48). In women, the fully adjusted model showed that age and population group remained significantly associated with low %BF. Mixed ancestry adolescents had higher odds of having low %BF compared with black adolescents (OR=2·48; 95 % CI 1·29, 4·76). Adolescents aged 18 years or above had higher odds of having low %BF than aged those less than 18 years (OR=2·21; 95 % CI 1·31, 3·74).

**Table 9:** Odds ratios and 95 % confidence interval for low &BF† from the adjusted logistic regression analyses in 18-year-old men and women, Johannesburg–Soweto, South Africa, Birth to Twenty Plus (Bt20+) cohort

		Step 1		Step 2	
	*n*	Adjusted OR‡	95 % CI	Adjusted OR‡	95 % CI
Men (*n* 550)					
Intrinsic factors					
Height	550	1·02	0·99, 1·05	1·03	0·99, 1·06
Age (<18 years§)	217				
≥18 years	333	1·68**	1·17, 2·41	1·74**	1·20, 2·51
Population group (black African§)	461				
White	35	0·55	0·26, 1·15	0·66	0·29, 1·52
Mixed ancestry	54	2·34*	1·19, 4·60	2·38*	1·20, 4·72
Low birth weight (<2500 g) (no§)	496				
Yes	54	1·60	0·84, 3·02	1·60	0·84, 3·02
Household socio-economic factors					
Caregiver education (3rd tertile (higher)§)	79				
≤Primary school	79			2·03^(^*^)^	0·99, 4·16
Secondary school	392			1·54	0·88, 2·71
Adolescent smoking status (never smoked§)	143				
Previous smoker	160			0·90	0·57, 1·44
Current smoker	247			1·60*	1·03, 2·48
Cox/Snell and Nagelkerke *R* ^2^ estimates		0·036, 0·049		0·057, 0·078	
Deviance		714·8		702·69	
Women (*n* 520)					
Intrinsic factors					
Height	520	1·04^(^*^)^	0·99, 1·08	1·04^(^*^)^	0·99, 1·08
Age (<18 years§)	219				
≥18 years	301	2·20**	1·30, 3·73	2·21**	1·31, 3·74
Population group (black African§)	439				
White	17	2·84^(^*^)^	1·00, 8·08	3·10^(^*^)^	0·92, 10·5
Mixed ancestry	64	2·40**	1·30, 4·41	2·48**	1·29, 4·76
Age of entry into menarche (<13 years§)	203				
≥13 years	317	1·64^(^*^)^	0·98, 2·73	1·63^(^*^)^	0·98, 2·72
Neighbourhood socio-economic factors					
Place of residence (Soweto§)	435				
Metropolitan Johannesburg	85			0·90	0·44, 1·86
Cox/Snell and Nagelkerke *R* ^2^ estimates		0·061, 0·101		0·061, 0·101	
Deviance		448·60		448·5	

%BF, percentage body fat. ^(^*^)^ *P*<0·10, *P<0·05, **P<0·01, ***P<0·001.

† Low %BF was defined as fat mass ≤15·00th percentile^(^ ^49^ ^)^.

‡ OR adjusted by logistic regression.

§ Reference category.

## Discussion

The purpose of the present research was to examine how both household and neighbourhood SEP influence BMI and %BF in South African adolescents and whether these associations differ by sex, to better understand the effect of the nutrition transition in this population.

### Anthropometric profile of adolescents

Our findings show that in adolescents, the pattern of nutrition transition differs between sexes. The 2012 South African NHANES^(^ ^22^ ^)^ showed that the prevalence of thinness in 15–17-year-olds was 26·2 % in men compared with 15·4 % in women. Contrastingly, the prevalence of overweight was 8·8 % in men and 27·3 % in women. In the current study, similar results were found, with a twofold higher prevalence of thinness observed in men compared with women (22·2 % *v*. 10·6 %). The proportion of overweight was approximately three times higher in women than in men (26·2 % *v*. 8·2 %). More women (24·0 %) had a high %BF than men (5·2 %). These findings are in line with the results of a systematic review conducted by Muthuri *et al*.^(^ ^54^ ^)^ on the overweight/obesity transition among children and adolescents in sub-Saharan Africa. That study showed the weighted average of overweight/obesity was higher among women compared with men. It also highlighted that undernutrition remained a concern in sub-Saharan Africa.

The overweight:thinness ratio was 0·37 in men and 2·47 in women in the present study. These figures represent a clear shift from thinness to overweight in women and also highlight the existence of a dual burden of malnutrition within the area of Johannesburg–Soweto, with high prevalence of thinness in men and high prevalence of overweight in women. This overweight:thinness ratio is likely to be even greater among adult women. Mendez *et al*.^(^ ^2^ ^)^ reported in 1998 that the overweight:thinness ratio among women aged 20–49 years was 14·2 in urban areas of South Africa. Although the overweight:thinness ratio is much lower in adolescents than in adults among women, this result suggests that the nutrition transition starts before adulthood in South African women. Earlier work with the cohort revealed little difference in mean BMI between boys and girls at the age of 9–10 years^(^ ^55^ ^)^.

### Influences of neighbourhood- and household-level socio-economic position on anthropometric status

The general pattern of association between SEP (measured at both the neighbourhood and household levels) and nutritional status in the current study is complex, with different associations observed for under- and overnutrition. In general, BMI and %BF were more often associated with household factors than with neighbourhood indices, especially for men; however, the associations were weak. A positive association was found between household SEP and overweight in men, while no household or neighbourhood SEP gradient was identified in women. These results are not in line with the results of reviews on overweight/obesity in LMIC, which showed an overall positive SEP gradient in obesity in youths^(^ ^16^ ^,^ ^54^ ^)^. The neighbourhood SEP environment, as measured in the present study, showed very little effect on the nutritional status of these adolescents and contrasts with evidence from high-income countries. A review from developed countries specifically studied the association between neighbourhood SEP (food, physical and built environments) in relation to weight status in youths and adults^(^ ^56^ ^)^ and found that a deprived neighbourhood in terms of economic and social resources was associated with high obesity rates in fifteen of the sixteen studies. This was supported by other reviews in children and adolescents assessing the association between the built environment and overweight/obesity^(^ ^57^ ^,^ ^58^ ^)^.

It is also important to mention that there were differences between findings using the conventional BMI measure and body fatness. BMI is widely used regardless of its limitations^(^ ^59^ ^)^. The importance of the neighbourhood social environment on women’s body fatness would not have been observed if the present study had focused only on BMI as a proxy for body fatness. Furthermore, if using BMI only, we would have concluded that there was a relationship between the household wealth index and thinness in women, when there was no association with low %BF. Finally, if using BMI only, we would also have concluded that there was an association between being a white man and overweight, when there was no association with high %BF. These findings underline that studies regarding SEP influences on anthropometric outcomes in adolescents should incorporate both fat mass and BMI measures.

### Sex differences in influences of neighbourhood- and household-level socio-economic position on anthropometric status

The pattern of association between SEP and nutritional status also showed clear differences between sexes.

In men, a positive household SEP gradient in overweight was observed. Those whose caregivers attained a secondary school level education *v*. a higher education level had significantly lower odds for overweight. A low household wealth index was associated with lower odds of being overweight and of having high %BF fat and with increased odds of being thin. These findings are in line with another study conducted in South African adults^(^ ^15^ ^)^ and with other studies conducted in LMIC which revealed a positive gradient between wealth and BMI/overweight in men^(^ ^14^ ^,^ ^60^ ^)^. Wrotniak *et al*.^(^ ^61^ ^)^ observed a positive association between SEP (type of school attended, asset ownership) and obesity in adolescents in Botswana. However, the men and women were pooled in the analysis. A study conducted by Bovet *et al*.^(^ ^62^ ^)^ in the Seychelles (part of the African region) also showed a positive relationship between SEP and overweight in men. Similar findings were reported in a review assessing overweight and obesity in children and youths in sub-Saharan Africa^(^ ^54^ ^)^. In terms of fat mass, previous work with a sub-sample of the Bt20+ cohort showed that high SEP children aged 9–10 years had higher fat mass than low SEP children^(^ ^55^ ^)^. The results of our study demonstrate that, in South African adolescent men, the shift of overweight from high to low household SEP groups has not begun. The high SEP group presents a higher risk for overweight and obesity in adolescent men and thus policy regarding non-communicable diseases should focus on wealthy and well-educated households. At the neighbourhood level, the odds of being overweight were increased for those in the lowest tertile of the neighbourhood economic index. This result was inconsistent with the household-level findings and thus warrants further investigation.

A different dynamic was observed in women. At the neighbourhood level, the odds of having a high %BF were increased for those in the middle tertile of the neighbourhood social support index compared with those in the third index tertile (favourable social environment). The pattern of increased risk in lower SEP groups, apparent in middle-income countries, is evident here^(^ ^10^ ^,^ ^12^ ^–^ ^14^ ^,^ ^17^ ^)^. The neighbourhood variables have been used previously in this cohort, relating SEP to anthropometric measures at 16 years^(^ ^36^ ^)^. This previous study found no neighbourhood SEP effect on anthropometric outcomes (although underweight was not investigated). The results found in the present study in relation to the neighbourhood social support environment suggest that, as the cohort transitions to a higher prevalence of overweight and obesity, the neighbourhood environment could begin to have more influence on anthropometric outcomes. The household SEP influences were minor for women. No SEP gradient in overweight was observed at the household level, similar to the findings of Alaba and Chola^(^ ^15^ ^)^. These results contrast with reviews on overweight/obesity in youths in LMIC^(^ ^16^ ^,^ ^54^ ^)^ and with a previous study on women aged 15–49 years in LMIC which showed that wealthy women had higher odds of being obese compared with their poor counterparts^(^ ^11^ ^)^. These results suggest that policy regarding non-communicable diseases should target all adolescent women regardless of their household SEP. The odds of being thin decreased for those in the lowest tertile of the household wealth index. This implies that thinness is less prevalent in the lowest SEP households and that the stage of nutrition transition is more advanced in adolescent women. Other influences contributing to thinness in women could be cultural factors such as body image perception and peer influences, which may differ by SEP^(^ ^63^ ^–^ ^67^ ^)^.

### Strengths and limitations of the study

To the authors’ knowledge, the present study is the first to look at household and neighbourhood SEP in relation to the dual burden of malnutrition in adolescent men and women and to focus on sex differences in the nutrition transition in urban South Africa. Furthermore, in the study both economic and social aspects of SEP have been examined, encompassing a wide range of SEP measures at the household and neighbourhood levels. This provides a more comprehensive assessment of SEP than most previous studies on this topic, which have focused on household SEP measures^(^ ^4^ ^,^ ^11^ ^–^ ^13^ ^,^ ^15^ ^,^ ^17^ ^,^ ^61^ ^)^. The Bt20+ cohort is the first to use a novel quantitative tool to measure self-perceived deprivation at the neighbourhood level among adolescents. This differs from previous studies which related child health outcomes to SEP data obtained mainly from national censuses and Demographic and Health Surveys, including measures such as employment, education, income and urban *v*. rural^(^ ^68^ ^)^. The use of convenient administrative boundaries to define neighbourhoods has been studied by Pickett and Pearl^(^ ^69^ ^)^ and Riva *et al*.^(^ ^70^ ^)^, with the former stating that boundaries ‘do not correspond to the actual geographical distribution of the causal factors linking social environment to health’^(^ ^69^ ^)^. Census data and household surveys do not take into account the social aspects of life and physical characteristics of the neighbourhood. Also, the residents’ perspective is lacking^(^ ^71^ ^)^. The tool used in the present study was based on previous formative work with the cohort^(^ ^42^ ^)^. In a recent review, van Vuuren *et al*.^(^ ^68^ ^)^ reported that, unlike the present study, a majority of studies (thirteen out of nineteen) assessing neighbourhood SEP and child health outcomes did not use theory-based neighbourhood constructs.

As the objective of the present study was to look at the influence of SEP at a single time point of adolescence (18 years), it is not possible to speculate as to potential underlying causal relationships. The lack of significance or consistent associations between neighbourhood SEP and the anthropometric outcomes could be explained by the neighbourhoods not varying enough to capture SEP-related differences. However, it could also result from the tool used to measure neighbourhood socio-economic deprivation. It is important to mention that the questionnaire developed is a general questionnaire designed to understand the nature of the socio-economic environments in which adolescents were residing. Further research using a more specific tool (including more refined measures, such as food environment, built environment and peer interactions^(^ ^72^ ^,^ ^73^ ^)^) to study nutritional outcomes, and adapted to urban settings in LMIC, may be required to clarify the associations between neighbourhood and health outcomes in adolescents.

Lifestyle factors (diet and physical activity) on the pathway between SEP and anthropometric outcomes need to be further investigated to guide policy.

In an attempt to adjust for the clustering of SEP characteristics within an area, multilevel modelling is often employed. However, the definition of neighbourhood used in the cohort resulted in no two households having the exact same neighbourhood and therefore no household shared exactly the same cluster, making the use of a multilevel structure unnecessary.

The current paper provides a greater understanding of the relationships between two levels of SEP in relation to nutritional status in adolescents living in this urban transitioning society. The study highlights that even within a relatively small urban area the nutrition transition manifests itself differently in men and women and across SEP indicators.

The fact that there are different problems affecting men and women within the same area adds complexity to the designing and implementation of appropriate health policies and makes the planning of public health services difficult. Understanding the challenges for different sexes at different ages is vital in helping to plan public health services.
